# Children and their families are entitled to the benefits of differentiated ART delivery

**DOI:** 10.1002/jia2.25482

**Published:** 2020-04-01

**Authors:** Lynne Wilkinson, George K Siberry, Rachel Golin, Benjamin R Phelps, Hilary T Wolf, Surbhi Modi, Anna Grimsrud

**Affiliations:** ^1^ International AIDS Society Cape Town South Africa; ^2^ Centre for Infectious Epidemiology and Research University of Cape Town Cape Town South Africa; ^3^ Office of HIV/AIDS United States Agency for International Development Washington DC USA; ^4^ Office of the U.S. Global AIDS Coordinator and Health Diplomacy Washington DC USA; ^5^ Centers for Disease Control and Prevention Atlanta GA USA

**Keywords:** HIV, ART, differentiated service delivery, children, stable, family‐centred

In 2017, the World Health Organization (WHO), together with the United States Centers for Disease Control and Prevention, the United States President's Emergency Plan for AIDS Relief (PEPFAR), the United States Agency for International Development and the International AIDS Society, recognized the importance of including children living with HIV who are clinically stable in differentiated antiretroviral (ART) delivery models to support family‐centred care [1]. As children living with HIV age and grow, ART dose adjustments become infrequent, with only three adjustments anticipated between the ages of one to seven years [1, 2]. Consequently, WHO’s *Key Considerations for Differentiated Antiretroviral Therapy Delivery for Specific Populations* outlined that children who are clinically stable are eligible for longer durations between ART refills and simplified drug pickup approaches. Clinical stability was defined as at least two years old, on ART for more than a year and the same regimen for at least three months, no adverse drug reactions requiring regular monitoring or current illness (including malnutrition), evidence of treatment success (two consecutive viral load measurements of <1000 copies/mL, rising CD4 counts or CD4 counts >200 cells/mm^3^) and caregivers orientated on the disclosure process. While viral load measurements are not required in this definition, it remains preferable, simplifying the assessment of clinical stability.

The WHO differentiates between children two to five years old and those older than five years who are clinically stable. Children over the age of five years – just like adolescents and adults – can space clinical consultations to every six months and ART refills to every three to six months. Children between two and five years old (“younger children”) can also benefit from longer intervals between clinical consultations and longer ART refills including three‐monthly ART refills and clinical consultations (rather than six‐monthly) [1]. In practice, this means that younger children only need to be seen four times annually for a simultaneous clinical consultation and ART refill.

Despite this guidance, many countries and providers have been hesitant to include children, especially younger children, in models that reduce their clinical consultation visit frequency [2, 3]. While many countries have differentiated service delivery (DSD) policies in place, children are often excluded. Where policies have included children, these have focused on children above the age of five years. Table 1 reflects national policies and guidance around children’s eligibility for differentiated ART delivery models. Only a few countries – Ethiopia, Ghana, Malawi and Zimbabwe – allow children who are clinically stable three‐monthly visit spacing but exclude younger children. To the best of our knowledge, only Mozambique and Namibia currently allow some enrolment in differentiated ART delivery models for younger children.

**Table 1:** Country policies regarding differentiated ART delivery eligibility for children

Country	Inclusion/exclusion criteria for children
Democratic Republic of Congo [4]	Stable patient definition applies to those over 15 years with no access to differentiated ART delivery models below this age
Eswatini [5]	Stable patient definition applied to adults over 18 years Adolescents 10 to 19 years specifically qualify for Teen Club model (monthly ART refills with six‐monthly clinical consultations)
Ethiopia [6]	Children zero to five years specifically excluded from stable patient definition but after six months on ART can be seen three monthly
Ghana [7]	Stable child defined as older than five years but allows children two to five years to be seen three‐monthly for clinical review with three‐monthly refill
Kenya [8]	Stable patient definition applies to those over 20 years but does allow classification of children and adolescents as stable stating “follow‐up appointment intervals beyond three months, when allowed, must take into consideration the need for weight‐based dose adjustments, close monitoring of support systems, and the stability of caregivers” Also states that child of stable caregiver/adult can be classified as stable if meets same criteria and qualify for differentiated care
Malawi [9]	Stable adults and children can be given three‐monthly appointments
Mozambique [10]	Stable patient definitions included for age groups two to nine years and above ten years Children two to nine years old qualify for three monthly clinical consultations and ART refills at the facility Children >10 years old qualify for six monthly clinical consultations and three monthly refills at the facility or through facility adherence clubs Children <15 years are not eligible for community ART groups.
Namibia [11]	Stable patient definition does not include age restriction and includes children in all differentiated ART delivery models with note to scrutinize weight and adherence to decide on frequency of visits
Nigeria [12]	Stable patients qualify for three‐monthly clinical visits and ART refills but children zero to five years excluded specifically requiring more regular monitoring
Rwanda [13]	Children under 15 years excluded from differentiated ART delivery models requiring monthly ART refills with three‐monthly clinical consultations
Sierra Leone [14]	Definition of stable child is over five years old
South Africa [15]	Stable patient definition only applies to adults over 18 years
Uganda [16]	Children qualify as stable at all ages but only eligible for facility‐based models. Children over five years qualify for three‐ monthly clinical consultations and ART refills, while under five years need to come monthly
Zambia [17]	Stable definition has no age restriction but unstable defined as “All children younger than five years old with HIV are considered as having advanced HIV disease”
Zimbabwe [18]	Children under two years should be seen monthly and thereafter three‐monthly until on adult doses

Country policies may have been influenced by WHO’s 2016 *Consolidated guidelines on the use of antiretroviral drugs for treating and preventing HIV infection* [19], where it was noted that DSD clinical stability inclusion criteria may not apply to “rapidly growing children and adolescents” [19], low viral load coverage among children [20] and WHO’s 2017 *Guidelines for managing advanced HIV disease and rapid initiation of antiretroviral therapy* [21], where all children under five years of age presenting to care were defined as having advanced HIV disease (AHD). The AHD guidelines specifically pertain to: (1) people presenting to care for the first time following an HIV diagnosis and (2) people who had previously started ART and are re‐engaging with care after a period of ART interruption. All children under age five years and not on ART were defined by the AHD Guideline Committee as having advanced disease based on heightened risk of disease progression and mortality, regardless of their clinical and immune condition, in the absence of prompt treatment. Varying age‐dependent CD4 cell count definitions for advanced immunosuppression also make definitions based on CD4 cell count difficult to implement in programmatic settings [22]. Importantly, AHD guidelines do not apply to children on effective ART – the group which can benefit from longer intervals between clinical consultation and drug pick‐up visits.

We believe that DSD policies could evolve to be more inclusive of younger age groups to ensure that new evidence and updated global guidance benefits all populations. In practice, caregivers in sub‐Saharan Africa commonly attend monthly facility visits on their child’s behalf without the presence of the child, resulting in a time and financial burden on the caregiver with no clinical benefit provided to the child. While younger children still need to visit the facility and see a clinician more frequently than their older siblings, parents or caregivers, differentiated ART delivery inclusion makes it more feasible to align family member visits and consider family member enrolment in the same model. Older family members can enrol in facility‐based individual models (such as multi‐month ART refills or fast track pre‐packed ART refill pick‐up) or group models (facility‐based adherence clubs or ART refill support groups) with the younger child’s visit dates *aligned* to specific model’s ART refills visit dates. Facilities can develop and implement differentiated ART delivery models that cater to both adults and their children. Effective, demonstrated models include facility‐based, fast track models providing quick, aligned ART refill pick‐up for all family members [23, 24] and family psychosocial support focused group models run at the facility (family ART adherence clubs) or in the community (community ART groups integrating children) [25, 26]. The family adherence club model could require younger children to see the clinician after every group visit until five years old and community ART groups could require younger children to attend the facility with the adult group member collecting ART refills on the group’s behalf every third month. These adaptations would enable younger children to be seen clinically every three months while simultaneously supporting family members to access the same differentiated ART delivery model. As has been stated previously, this family‐centred approach is critical to ensuring that family members benefit from differentiated ART delivery, benefits that are lost if parents are unnecessarily required to take stable younger children to attend clinics more frequently [2, 27].

In conclusion, we want to draw attention to the WHO endorsement of the inclusion of children living with HIV who are clinically stable on ART, including children aged two to five years, in differentiated ART delivery models. To facilitate adherence among all family members living with HIV to lifelong ART, countries should urgently consider revising DSD policies, guidelines and/or standard operating procedures to specifically include eligibility criteria for children above two years old and prioritize viral load for children. Facilities can then focus on improving family members clinical consultation and ART refill visit alignment and consider enrolment of family members in the same differentiated ART delivery model thereby providing true family‐centred care.

## Competing interests

None of the authors have competing interests to declare.

## Authors’ contributions

The concept for this commentary was developed by LW, AG, GS, RG, BRP, SM and HW. LW wrote the first draft. All authors contributed and approved the final version.
